# Effect of Zinc Excess in Substrate on Physiological Responses of *Sinapis alba* L.

**DOI:** 10.3390/plants12010211

**Published:** 2023-01-03

**Authors:** Natalia Repkina, Irina Nilova, Natalia Kaznina

**Affiliations:** Institute of Biology, Karelian Research Centre, Russian Academy of Sciences, 11 Pushkinskaya St., 185910 Petrozavodsk, Russia

**Keywords:** white mustard, heavy metal, photosynthesis, zinc concentration, phytoremediation

## Abstract

Zinc (Zn) is a fundamental micronutrient for plants’ metabolism, but in high concentrations, it is toxic. In this study, we investigated the physiological response of white mustard (*Sinapis alba* L. cv. Belgia) plants to the Zn excess concentrations (50, 100, and 150 mg kg^−1^) in the substrate. The results showed that sand Zn concentration of 50 mg kg^−1^ did not affect the physiological parameters of plants, despite to the high Zn accumulation in shoots. The growth, biomass accumulation, photosynthesis rate, and pigment amount were inhibited at Zn concentrations of 100 and 150 mg kg^−1^ in substrate. A slight increase in malondialdehyde (MDA) was also observed at zinc concentrations (100 and 150 mg kg^−1^) without changes in membrane permeability, which is partly connectedtoan increase in the proline content. The results suggested that white mustard tolerates Zn excess impact. *S. alba* is able to grow on Zn-contaminated substrates along with significant Zn accumulation in shoots, which supports its high potential for phytoremediation of Zn-polluted agricultural soils. It is also possible to propose the following recycling of white mustard plants for Zn fortification feedstuff.

## 1. Introduction

The contamination of the agriculture soils with pollutants is one of the reasons for a decrease in soil quality and crop production [1,2]. Among the pollutants, heavy metals are one of the most hazardous materials [1]. Even at low concentrations, heavy metals are able to keep the toxic effect on plants and through the food chain uptake by animals and humans [3]. Heavy metals include 53 elements with atomic mass more than 5 Da and density >5 g cm^−3^, and based on their requirements in metabolism, they can be separated into essential and non-essential elements [1]. Zn is an essential element, which in high concentrations can be toxic for plants [4,5]. Zinc plays a crucial role in metabolism of all the living organisms [4,6], being one of the main structural components of Zn-depending proteins [4]. Along with its biological role as a regulatory ion and as an intracellular second messenger, Zn participates in membrane integrity and stabilization, partly due to the alleviation of oxidative stress [7]. Additionally, Zn is responsible for regulation of chromatin structure, RNA metabolism, and protein–protein interactions through effects on DNA/RNA binding and site-specific modifications [8,9].

The natural source of Zn in the soils is weathering of parent materials [10]. However, anthropogenic sources, such as extraction of commercial minerals, founding, large-scale industry, energy and fuel production, energy transmission, and intensive agriculture, are the main contributors to Zn pollution [11]. According to this, the search for eco-friendly approaches and technologies that allow for cleaning contaminated soils is currently important [12]. One of the natural approached with great potential technology is phytoremediation [13]. Remarkably, the realizing of phytoremediation technology is cost-effective, due to its independence from highly qualified personnel or expensive equipment [14]. There are several strategies of phytoremediation, but two of them are more effective: phytoextraction—the absorption of metals from soil by metal accumulators and sequestrating them in the aboveground part; and phytostabilization—process by which plants stabilize the metal ion by roots, resulting in a drop of pollution in soil [15,16].

However, limited plants species are suitable for phytoremediation. There are some criteria for plants for being selected to carry out the process of phytoremediation, such as root depth, soil contaminants, soil and regional climate, etc. [12]. Moreover, for successful phytoremediation process, the plants should be responsible for high accumulation of metals and resistant to heavy metal influence. According to this, the search for plant species with high phytoremediation potential is a current concern. In recent years, high interest has been paid to the *Brassicaceae* family, some members of which are able to accumulate a significant amount of Zn, with the following among them: *Thlaspi caerulescens* [17,18], *Capsella bursa-pastoris* [19,20], *Brassica juncea* [21,22], and *Brassica napus* [23,24].

*B. juncea* is esteemed as a hyperaccumulator as *B. juncea* is one of the perspective plant species of the *Brassicaceae* family for phytoremediation of contaminated soils [16]. The mechanisms of tolerance to heavy metals that allow for this species to grow successfully on soils with high Zn concentration are described. Between the transport ions in the aboveground parts of plant, there are binding ions by chelators in cytoplasm with further transport in vacuole. As opposed to *B. juncea*, the *Sinapis alba* L. (white mustard) is not a hyperaccumulator, but single datum demonstrated its capability to accumulate significant amount of metals, including Zn. For instance, *S. alba* accumulated around 3000 mg g^−1^ dry weight in shoots with added Zn concentration (300 µM) in substrate [25]. Moreover, under this condition, *S. alba* not only kept growing, but also rich the stage of seed germination. Some authors supposed *S. alba* as a potential Zn accumulator and good candidate for phytoremediation of Zn-contaminated soils [26]. Additionally, there are data that show the drop in Zn concentration into the substrate after growing *S. albaon* them, which suggest its potential in remediation of contaminated substrates [27].

*S. alba* is a valuable agricultural crop and uses as a raw material for the production of oil and spices as well as green manure [28]. Taking this into account as well as its capability to accumulate high concentrations of Zn aboveground, *S. alba* can be recommended for nutrient fortification of animal feedstuff. This opportunity can help to solve the double problem: on the one hand, the remediation of Zn-contaminated agricultural soils during growing of white mustard, and on the other hand, further recycling of these plants for fortification of animal feedstuff, as an example. Additionally, there are data that show that *S. alba* is more resistant to several stressors, including heavy metals, in comparison with other species of *Brassicaceae* [29]. Further, *S. albais* characterized by a faster and deeper root system [30]. Overall, it is possible to suppose that *S. alba* is available for phytoremediation with further recycling. However, for confirmation, we need more investigation of the responses and physiological reactions of plants to Zn excess, which are still poorly studied.

Accordingly, in the present study, Zn accumulation and physiological parameters were investigated in white mustard grown in Zn-contaminated substrate.

## 2. Materials and Methods

### 2.1. Plant Material, Growth Conditions, and Treatments

Mustard seeds (*Sinapis alba* L. cv. Belgia) were acquired from the Federal Research Center N. I. Vavilov All-Russian Institute of Plant Genetic Resources (VIR), Ministry of Science and Higher Education, Russia. This study was conducted at the research base of the Agricultural Experiment Station (61°45′6″ N, 119°25′10.9″ E) (WGS84), Petrozavodsk, Republic of Karelia, Russia. Mustard seeds sown 1 day prior were steeped in distilled water. Two-day-old seedlings (12 seedlings per a pot) were placed in plastic pots (1 L). Each pot contained 0.8 kg of pure quartz sand that previously was washed and sterilized. Appropriate amounts of sulphate salt of Zn (ZnSO_4_·H_2_O) were added once to the sand to maintain the required levels of Zn: 5 (control), 50, 100, and 150 mg kg^−1^ of substrate (in terms to ions of metal). Before the sown seeds, the sand in the pots was mixed in order to ensure uniform distribution of element. During the growing period, pots were irrigated by Hoagland–Arnon nutrient solution excluding the addition of Zn. All pots were placed in a greenhouse under natural day/night conditions with day/night temperature of 21/14 ± 2 °C and 77 ± 5% relative humidity during the growing period. The experimental design was completely randomized with three replications. Plants were harvested and analyzed after 3 weeks (stage of 3–4 true leaves).

### 2.2. Determination of Zinc Content 

An inductively coupled plasma-mass spectrometry (ICP-MS) (Agilent 7900, Santa Clara, CA, USA) analysis was performed to determine the concentration of Zn in roots and shoots of white mustard. The samples were digested by microwave prior to analysis, using the Speedwave Digestion system (Berghof, Eningen, Germany). The extraction was performed in a solution of nitric acid.

### 2.3. Analysis of Growth-Related Parameters

*S. alba* seedlings were separated into shoots and roots, further rinsed using distilled water, and dried on filter paper for fresh weight (FW) detection. Dry weight (DW) was evaluated after drying the shoots and roots to a constant weight at 80 °C. The relative water content (RWC) was estimated in percentage (%) using the following formula: RWC = ([FW − DW]/FW) ∗ 100, where FW is the fresh weight and DW is the dry weight

### 2.4. Photosynthetic Pigment Content Measurement

Photosynthetic pigments, including chlorophyll *a*, chlorophyll *b*, total chlorophyll, and carotenoids were analyzed spectrophotometrically on SF2000 spectrophotometer (Spectrum, Moscow, Russia) [31]. For the extraction of chlorophyll pigments, the 85% (*v*/*v*) aqueous acetone was used. The absorbance of the supernatant was measured at 665, 649, and 440.5 nm alongside a blank of untainted 85% liquid acetone. Photosynthetic pigments’ concentration was analyzed using known formulas. Considering that almost all Chl*b* was located in light harvesting complex II (LHCII) and that the ratio of Chl*a* and Chl*b* in LHCII was 1.2, the percentage of Chl in LHCII was calculated [32].

### 2.5. Gas Exchange and Chlorophyll a Fluorescence Assay

At least five fully-grown first true leaves per treatment were used for the measurement of chlorophyll *a* fluorescence using a portable chlorophyll fluorimeter MINI-PAM (Walz, Effeltrich, Germany). After 20min dark adaptation conditions, the maximum efficiency (F*_v_*/F*_m_*) of photosystem II (PSII) was measured. The following parameters of gas exchange were obtained: stomatal conductance (G_s_), net photosynthetic rate (P_n_), and transpiration rate (E). All parameters were analyzed on the first fully developed leaves using portable photosynthesis system HCM-1000 (Walz, Effeltrich, Germany). The measurements were performed under a CO_2_ concentration of 400 µmol^−1^ and a photon flux density of 1200 µmol m^−2^ s^−1^. The cuvette temperature was held at 25 °C at a relative humidity of 60–65%. Water-use efficiency (WUE) was calculated by dividing the net photosynthetic rate by the transpiration rate.

### 2.6. The Free Proline Content

The proline content was analyzed by the Bates et al. (1973) method [33]. First, 0.5 g of fresh mustard leaves were processed by homogenizing in 10 mL of 3% sulfosalicylic acid at 5100× *g* for 5 min. Further, 2 mL ninhydrin reagent and 2 mL glacial acetic were added to 2 mL of supernatant. All test tubes were placed in water bath for 1 h at 100 °C, followed by a stop reaction by cooling in an ice bath. The absorbance of the mixture was measured at 520 nm. The standard curve of L-proline was used for determining the proline concentration.

### 2.7. Determination of Lipid Peroxidation

The measurement of malondialdehyde (MDA) content was conducted according to the method of Stewart and Bewley (1980) [34]. The mustard leaves (0.1 g) were mixed with 2 mL 5% thiobarbituric acid (TBA) in 20% trichloracetic acid (THA) and then centrifuged at 10,000× *g* for 15 min at 4 °C. All tubes were heated in water bath at 95 °C for 30 min, followed by cooling in an ice bath. Then, solutions were again centrifuged at 10,000× *g* for 5 min. The absorbance of the supernatant was measured at 532 and 600 nm. The MDA content was calculated using an extinction coefficient of 155 mM^−1^cm^−1^.

### 2.8. The Relative Electrolyte Leakage (REL) Assay

REL from the leaves tissues was determined to estimate the membrane permeability. Fresh leaf discs (3 g) of mustard were swill with distilled water and then dried with filter paper. Then discs were infiltrated and placed in glass flask and mixed with 30 mL of distilled water. All flasks were shaken for2 h at 23 °C, with further measurement of electric conductivity of the solution (E1) by a conductometer («HANNA», Villafranca, Padovana, Italy). Then, all flasks were heated until boiling, cooled to room temperature, and the full electrolyte leakage (E2) was determined. REL (%) was calculated by the formula REL = 100 − E1/E2.

### 2.9. Statistical Analysis

Statistical analysis was performed by using Excel 2007 (Microsoft Corp., Redmond, WA, USA) and Statgraphics Plus 5.0 (Statgraphics Technologies, Inc., The Plains, VA, USA) statistical software. Results are shown as mean ± standard error (SE). Analysis of variance (ANOVA) was applied to the resulting data. Fisher’s least significant difference (LSD) test was used to detect the statistically relevant differences at *p* ≤ 0.05.

## 3. Results

### 3.1. Effect of Zn Excess on Zn Content in S. alba Seedlings 

Analysis of the zinc content in *S. alba* plants showed that Zn concentration in plants increased along with the increase in Zn concentration in the substrate (Table 1). At the same time, the amount of Zn in roots and shoots was almost equal at all studied concentrations.

### 3.2. Effect of Zn Excess on Shoot Growth and Biomass Accumulation of S. alba Seedlings 

All studied growth parameters remained unaffected by the Zn concentration of 50 mg kg^−1^ (Table 2). The growth of mustard was inhibited by the influence of Zn concentrations (100 and 150 mg kg^−1^) in substrate. An increase in Zn concentration up to 100 and 150 mg kg^−1^ of substrate led to a decrease in shoot height (by 2- and 4-fold, respectively), shoot FW (by 3- and 5-fold, respectively), and DW (by 2- and 3-fold, respectively) (Table 2).

### 3.3. Effect of Zn Excess on Photosynthetic Apparatus (PSA) of S. alba Seedlings 

The content of zinc at a concentration of 50 mg kg^−1^ in the substrate did not affect the PSA of plants. In contrast, the increase in Zn concentrations in substrate of up to 100 and 150 mg kg^−1^ caused a decrease in photosynthetic pigment content (Table 3). The amount of chlorophyll *a* and carotenoids declined by about 30%, whereas the amount of chlorophyll *b* in LHCII content dropped by 50% compared to the control under a Zn concentration of 100 mg kg^−1^. The Zn substrate concentration of 150 mg kg^−1^ had a stronger effect on all the studied parameters, resulting in a decrease by 50% compared to the control. 

**Figure 1:**
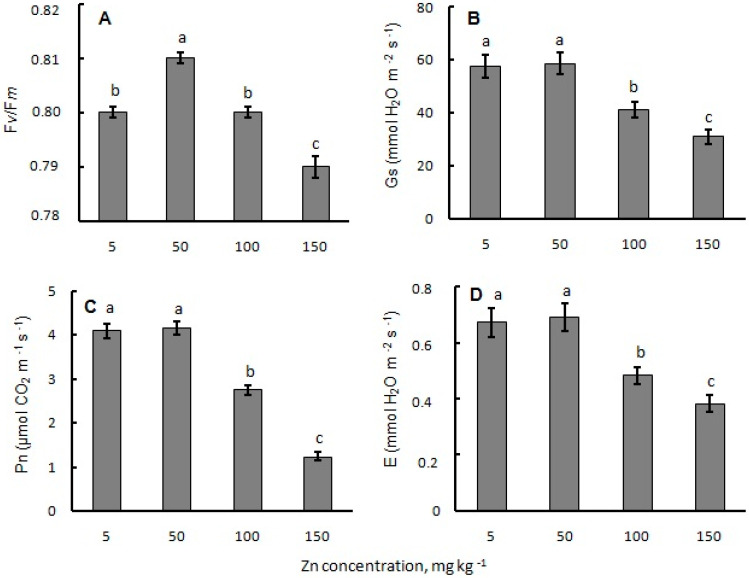
Effect of Zn excess in substrate on (**A**) maximum efficiency of PSII photochemistry (F*_v_*/F*_m_*), (**B**) stomatal conductance (Gs), (**C**) net photosynthetic rate (Pn), and (**D**) transpiration rate of white mustard plants. Data are represented as mean ± SE (*n* = 18). Data followed by the same letter are not significantly different at *p* < 0.05 (ANOVA).

The value of the F*_v_*/F*_m_* index was slightly higher in mustard plants grown under the Zn concentration of 50 mg kg^−1^ in substrate compared to the control (Figure 1A). The Zn substrate concentration of 100 mg kg^−1^ did not affect this parameter, while the highest concentration (150 mg kg^−1^) led to a significant decrease in the F*_v_*/F*_m_* parameter. 

All studied photosynthetic parameters—stomatal conductance, net photosynthetic rate and transpiration rate—remained unaffected under 50 mg kg^−1^ Zn in the substrate (Figure 1B–D). All these parameters decreased 1.5-fold in mustard seedlings at 100 mg kg^−1^ of Zn concentration in the substrate. Under 150 mg kg^−1^ of external Zn concentration, the stomata conductance and net photosynthetic rate declined compared to the control by about 50% (Figure 1B,C). Under the Zn concentration of 150 mg kg^−1^, the transpiration rate decreased less than the photosynthesis process, but its level was lower in comparison with other studied Zn concentrations (Figure 1D).

### 3.4. Effect of Zn Excess on Water Content of S. alba Seedlings 

An almost double decrease in WUE was observed in white mustard under the 150 mg kg^−1^ Zn concentration in the substrate compared with the control, while under lower Zn concentrations, this parameter did not change (Table 4). Relative water content (RWC) declined at the Zn concentrations of 100 and 150 mg kg^−1^ (Table 4). The Zn concentration of 50 mg kg^−1^ did not affect this parameter.

### 3.5. Effect of Zn Excess on Relative Electrolyte Leakage (REL), Malondialdehyde (MDA), and Proline Content in S. alba Seedlings

The EL remained unaffected under all studied Zn concentrations (Table 5). The MDA level increased at Zn concentrations of 100 and 150 mg kg^−1^ in the substrate (Table 5). The effect of both concentrations on MDA content was similar. Despite the absence of an effect on the MDA level by 50 mg kg^−1^ Zn concentration, its effect caused an increase in proline accumulation compared to the control (Table 5). Higher concentrations of zinc (100 and 150 mg kg^−1^) also resulted in rise of proline content in mustard plants (Table 5).

## 4. Discussion

Heavy metal pollution of soil has become a global environmental challenge due to intensively increasing industrialization and agricultural activities. Sources of heavy metals in soil include excessive application of agrochemicals, sewage sludge, industrial wastewater, biosolids, and manure [35,36]. Heavy metals are grouped into essential and non-essential classes. Essential metals, including Zn, are micronutrients but become toxic when taken in excess quantities [37,38,39]. Therefore, much attention has been paid to the development of various methods and technologies for cleaning and restoring zinc-contaminated areas, and among them is the phytoremediation method [13]. However, the effectiveness of this approach depends on the tolerance of plants to great concentrations of Zn in soils [40]. Taking this into account, the plant species belonging to the *Brassicaceae* family seem to be good candidates for phytoremediation [41,42].

Among plants of the *Brassicaceae* family, the *B. junceae* as a species with high tolerance to the effects of Zn contamination has been well investigated. This plant species, described as a hyperaccumulator, and its main mechanisms of tolerance have been studied [16]. *B. junceae* is recommended as one of the perspective plant species for the phytoextraction approach to polluted soils. At the same time, *S. alba* has not been described as a hyperaccumulator, but it has been successfully growing on soils with a zinc concentration of 1000 mg kg^−1^ [26], accumulating its high concentration aboveground. However, in contrast to *B. junceae*, the *S. alba* mechanisms of tolerance as well as its physiological reactions are poorly described.

In the present study, the capability of white mustard to accumulate significant amounts of Zn in the under- and aboveground parts of plants at its excess in substrate was detected. Meanwhile, the Zn content in above- and underground parts was similar. These data corresponded with the data of Soleimannejad et al., 2020 [26]. The increase in Zn accumulation by plants seems to be the one of the main reasons for physiological process inhibition under Zn excess influence. Almost all physiological parameters were not affected by the Zn concentration of 50 mg kg^−1^, whereas, under the Zn concentrations 100 and 150 mg kg^−1^, these parameters were inhibited. Particularly, the Zn excess hinders the growth of mustard, which was also described by Stanislawska-Glubiak and Korzeniowska, 2011 [43] under zinc addition of 200 and 400 mg kg^−1^ into the soil. The negative effect of Zn excess was also observed in other species of the *Brassicaceae* family: in *B. oleracea* [44], *B. napus,* and *B. juncea* [45].This suggests that growth inhibition under Zn excess concentrations can be a result of limiting in cell division due to an increase in the duration of mitotic phases as well as the full mitotic cycle [46] and impaired cell elongation due to a decrease in the elasticity of cell walls or a decrease in their turgor [47]. Besides, the drop in the other physiological processes, including photosynthesis, water regime, and mineral nutrition, could also be a reason. At the same time, inhibition of plant growth under stress conditions may be an adaptive response aimed at conserving the resources to increase the plant’s tolerance. The productivity of plants under stress conditions depends on the ability of plants to maintain photosynthesis activity and maintain the required level of water metabolism. However, in the available literature, there are almost no data that describe the effect of excess zinc on the process of photosynthesis in white mustard. Mostly, the results presented describe changes in the content of photosynthetic pigments. Commonly, Zn excess leads to a decrease in pigment content caused by a drop in the rate of photosynthesis [48,49,50,51].

Previously, it was shown that pigment content in parallel with chlorophyll content in LHCII in *S. alba* dropped under substrate Zn concentrations of 100 and 150 mg kg^−1^ [50,51]. However, the absence of significant changes in pigment content in white mustard were shown, even at increase in Zn concentration in soil up to 1000 mg kg^−1^ [26]. In the present study, a decline in chlorophyll and carotenoid content in leaves of white mustard at substrate Zn concentrations of 100 and 150 mg kg^−1^ was also demonstrated in parallel with a drop in chlorophyll content in LHCII that caused the negative effect on light absorption.

Maintaining a high level of photosynthesis is directly related to the reactions occurring in the light phase of the CO_2_ assimilation process [52]. It is known that, in healthy leaves, the change of maximum quantum yield of chlorophyll fluorescence (F*_v_*/F*_m_*) is usually ranges from 0.75 to 0.84 [53]. The absence of the inhibitory effect of excess Zn concentrations on the value of the F*_v_*/F*_m_* index indicated a high efficiency of photosystem II under metal stress (Figure 1A). These data, with respect to the results, can be achieved on other plant species [54,55].

Under high Zn concentrations (100 and 150 mg kg^−1^) in white mustard, we observed a decrease in stomatal conductance. It is known that metals cause stomatal closure, changes in the membrane permeability of guard cells, and a sharp increase in the level of abscisic acid, leading to partial or complete prevention of the stomatal opening [56]. However, this effect can be a result of the direct negative impact of Zn on membrane structure and permeability as well as on the destruction of guard cells [57]. Further, this plant reaction can be necessary for keeping the relative water content of tissue. According to this, the absence of a strong decrease in the water content of white mustard and partial stomata closure connecting to the drop of the transpiration rate are an adaptive mechanism aimed at maintaining the water content in the plants [58].

According to the data, the successful adaptation of plants to metal stress depends on water efficiency during photosynthesis, which allows the plant organism to increase resources following synthesis of plastic substances for growth and biomass accumulation [59]. The decline of WUE was observed only under Zn concentration 150 mg kg^−1^ in substrate, which can be result of disrupting the balance of CO_2_/H_2_O gas exchange processes [60]. However, WUE change was not as intensive as changes in the photosynthesis process. Particularly, the photosynthesis double dropped under the Zn concentration of 150 mg kg^−1^ due to partly to stomatal closure and a decrease in photosynthetic pigment content and disturbance light absorption.

It is well known that the inhibitory effect of Zn on physiological processes can be associated with the destruction of cell membranes [61]. High concentrations of metals increase the permeability of cell membranes, cause a sharp increase in reactive oxygen species (ROS), and cause an increase in the intensity of lipid peroxidation [62,63]. However, the membrane permeability of white mustard remained unaffected even at high Zn concentrations, despite an increase in MDA level as a marker reaction of lipid peroxidation at 100 and 150 mg kg^−1^ of Zn concentrations in substrate. To protect themselves against developing oxidative stress, plants have an effective antioxidant defense system including enzymatic and non-enzymatic compounds [64]. The increase in activity of the main antioxidant enzymes in white mustard [26], rice [65], wheat [66], barley [67], etc. under Zn excess was demonstrated. Along with enzymatic components’ activation, the non-enzymaticproline accumulation was investigated [68]. Proline, as a multifunctional amino acid, has diverse roles in stress protection, such as stabilization of proteins, stabilization of subcellular structures and membranes, and scavenging ROS [69,70]. The involvement of proline in the detoxification of the hydroxyl radical and superoxide radical has been shown previously [71]. According to proline multifunction, its increase under Zn excess can describe the absence of changes in membrane permeability. Generally, the activation of all components of antioxidant system also can be seen as one of the successful mechanisms for the efficient prevention of lipid peroxidation, ROS accumulation, and membrane damage that along with other physiological changes promote white mustard tolerance to Zn excess.

## 5. Conclusions

White mustard accumulates a significant amount of Zn in under- and aboveground parts. The Zn concentration in shoots is 2500 ppm at 50 mg kg^−1^ in substrate, which did not affect the physiological processes and germination. Accumulation of Zn of more than 2500 ppm in shoots (Zn concentration in substrate is 100 and 150 mg kg^−1^) caused a decrease in photosynthetic pigments content, light absorption, and partial closure of stomata, which is the reason for a drop in the photosynthesis rate following the decline of dry weight accumulation. Despite these changes, the white mustard is able to grow and reach the seed maturation stage. This is an option through the activation of physiological and biochemical mechanisms of tolerance, such as growth impairment, a decrease in transpiration resulting in maintaining the water relative content, promotion of photosystem II activity, and an increase in proline content that partly supports keeping the ROS balance and membrane permeability. Taking into account the capacity of white mustard to grow under high Zn concentrations in substrate and maintain yield capacity along with accumulation of Zn in high concentrations in the shoots, it is suggested that white mustard is characterized by high potential for phytoremediation with further recycling in the fortification of feedstuff.

## Figures and Tables

**Table 1:** Effect of Zn excess on Zn accumulation in shoots and roots of *S. alba* seedlings.

Zn Concentration, mg kg^−1^ Substrate	Zn Content, ppm	
	Shoot	Root
5	187.32 ± 20.60 d	115.01 ± 14.80 e
50	2505.31 ± 206.02 c	1504.60 ± 125.96 c
100	2620.35 ± 215.22 b	2708.28 ± 222.26 b
150	3118.52 ± 311.08 a	3340.07 ± 272.80 a

Data are represented as mean ± SE (*n* = 27). Data followed by the same letter are not significantly different at *p* < 0.05 (ANOVA).

**Table 2:** Effect of Zn excess on *S. alba* seedlings height, fresh (FW), and dry weight (DW).

Zn Concentration, mg kg^−1^ Substrate	Shoot Height, cm	Shoot FW, g	Shoot DW, g
5	26.22 ± 1.84 a	1.27 ± 0.14 a	0.11 ± 0.01 a
50	23.49 ± 1.26 a	1.36 ± 0.15 a	0.11 ± 0.01 a
100	11.66 ± 0.51 b	0.46 ± 0.04 b	0.05 ± 0.01 b
150	6.94 ± 0.48 c	0.27 ± 0.03 c	0.04 ± 0.01 c

Data are represented as mean ± SE (*n* = 27). Data followed by the same letter are not significantly different at *p* < 0.05 (ANOVA).

**Table 3:** Effect of Zn excess on photosynthetic pigment content in *S. alba* seedlings.

Zn Concentration, mg kg^−1^ Substrate	Chlorophyll *a*, mg g^−1^ FW	Chlorophyll *b*, mg g^−1^ FW	Total Chlorophyll (*a*+*b*), mg g^−1^ FW	Carotenoids, mg g^−1^ FW	Chlorophyll Content in LHCII, %
5	0.98 ± 0.03 a	0.38 ± 0.02 a	1.36 ± 0.04 a	0.28 ± 0.01 a	0.18 ± 0.02 a
50	0.94 ± 0.03 a	0.41 ± 0.01 a	1.35 ± 0.04 a	0.25 ± 0.01 a	0.20 ± 0.01 a
100	0.62 ± 0.02 b	0.26 ± 0.01 b	0.88 ± 0.03 b	0.18 ± 0.01 b	0.09 ± 0.01 b
150	0.38 ± 0.02 c	0.16 ± 0.01 c	0.53 ± 0.03 b	0.13 ± 0.01 c	0.03 ± 0.01 b

Data are represented as mean ± SE (*n* = 27). Data followed by the same letter are not significantly different at *p* < 0.05 (ANOVA).

**Table 4:** Effect of Zn excess on water content in *S. alba* seedlings.

Zn Concentration, mg kg^−1^ Substrate	WUE, µmol CO_2_ mmol H_2_O^−1^	RWC, %
5	6.32 ± 0.44 a	91.37 ± 0.17 a
50	6.39 ± 0.61 a	91.73 ± 0.18 a
100	5.84 ± 0.19 a	89.44 ± 0.31 b
150	3.34 ± 0.24 b	86.44 ± 0.45 c

Data are represented as mean ± SE (*n* = 27). Data followed by the same letter are not significantly different at *p* < 0.05 (ANOVA).

**Table 5:** Effect of Zn excess REL, MDA, and proline content in *S. alba* seedlings.

Zn Concentration, mg kg^−1^ Substrate	REL, %	MDA, nmol g^−1^ FW	Proline, mmol g^−1^ FW
5	10.80 ± 0.83 a	1.98 ± 0.09 b	6.11 ± 0.30 b
50	11.30 ± 0.77 a	2.10 ± 0.08 b	16.40 ± 1.97 a
100	11.00 ± 1.23 a	2.77 ± 0.15 a	22.95 ± 5.59 a
150	12.30 ± 0.06 a	2.67 ± 0.19 a	17.53 ± 3.93 a

Data are represented as mean ± SE (*n* = 12). Data followed by the same letter are not significantly different at *p* < 0.05 (ANOVA).

## Data Availability

All data, tables, and figures in this manuscript are original.
